# Role of visual and olfactory cues in sex recognition in butterfly *Cethosia cyane cyane*

**DOI:** 10.1038/s41598-017-04721-6

**Published:** 2017-07-10

**Authors:** Chengzhe Li, Hua Wang, Xiaoming Chen, Jun Yao, Lei Shi, Chengli Zhou

**Affiliations:** Key Laboratory of Cultivating and Utilization of Resources Insects of State Forestry Administration, Research Institute of Resources Insects, Chinese Academy of Forestry, Kunming, 650224 Yunnan China

## Abstract

Butterflies use multiple signals, including visual, olfactory and tactile cues, to identify same- and opposite-sex individuals during courtship. In this study of the sexually dimorphic butterfly *Cethosia cyane cyane*, we explored the roles of visual and olfactory cues in conspecific mate recognition during courtship. Our results showed that males took the initiative in actively chasing females during courtship using only visual cues. Males could distinguish the gender of conspecifics using visual cues alone. The size and color of the wings differ significantly between the sexes. Behavioral assays showed that males visually recognized females not by wing size, but by their sexually specific wing color. The movement pattern of the model also exerted some influence on male courtship chasing behavior. A total of 21 volatiles were detected in the bodies of adults, but only cedrol played a role in the process of male recognition of females at close range. Therefore, males rely on both visual and olfactory cues to distinguish females during courtship. Visual cues play a major role in attracting males at the beginning of the courtship chase, while olfactory cues play a role in accurately identifying partners at close range.

## Introduction

The courtship behavior of butterflies poses many interesting ecological questions. An understanding of courtship behavior is essential to any attempt to evaluate the evolution of sexual dimorphism and the selective consequences of variation between the sexes^1^. The behavior of males and females during courtship chases provides clues regarding the kind of information used to identify same- and opposite-sex individuals. In a variety of butterflies, the males find females visually and take the initiative in chasing the female^2, 3^. Sexual communication in butterflies involves the use of multiple signals at different stages of the mate recognition and choice process, resulting in a complex scenario. Males fly around in suitable areas in search of females, and approach any females encountered. It is well known that individual butterflies identify conspecific mates using sex-specific visual, olfactory, tactile, or other cues^4–6^.

However, different butterfly species use different signals for identifying conspecific mates. Many species use the differences in UV reflectivity between male and female wings for conspecific mate recognition e.g. *Pieris protodice* (Pieridae)^7^, *Colias eurytheme* Boisduval and *C. philodice* Godart (Pieridae)^8^. Some butterfly wings reflect little or no UV light, and sexual differences in wing size or color enable conspecific mate recognition using visual cues. Both sexes of *Pieris rapae rapae* L. (Pieridae) reflect low levels of UV light^9^; males and females have different markings on the dorsal surface of the forewings so that males can readily discriminate between the sexes visually^3^. *Dryas iulia* Fabricius (Nymphalidae) is sexually dimorphic both in wing size and coloration, with males being more colorful and larger than the females^10^. Visual patterns appear to be important in choosing and recognizing sexual partners^1^. Although some butterfly species have wings which reflect UV light, some show no difference between males and females, and sexual discrimination in these species depends mainly on olfactory cues e.g. *Pieris melete* Ménétriès (Pieridae)^11^. The reflectivity pattern of the wing undersurface is approximately similar in both sexes in *Zizeeria maha argia* (Lycaenidae)^12^, and males distinguish females from males using olfactory cues^13^. The absence of color dimorphism in species of the *februa* species group (Nymphalidae) suggests that they use chemical signals to determine gender^14^. Butterflies with sexual color dimorphism are supposed to recognize each other visually^15^, but there is little evidence for this.

Our study compares the roles of visual and olfactory cues during courtship in Lepidoptera, using the sexually dimorphic butterfly *Cethosia cyane cyane* Drury (Lepidoptera: Nymphalidae). Previous studies of *C. cyane cyane* have focused mainly on their morphology and biology^16, 17^, population life table in captivity^18^, visual and olfactory responses during foraging^19^, and biogeography and systematics^20^. However, there is little information in the literature on this species regarding the cues that males use to identify opposite-sex individuals during courtship.

The aim of this study was: (1) to evaluate whether males rely on wing size and color differences between the sexes to identify females; and (2) to examine whether olfactory cues are involved in the identification of females, and if so, what compounds are involved? Analyzing the visual and olfactory cues used by male *C. cyane cyane* should help to deepen our understanding of sexual interactions, and the relative importance of color and odor in mate recognition in the sexually dimorphic butterfly.

## Results

### Courtship behavior

#### Natural population

**Figure 1:**
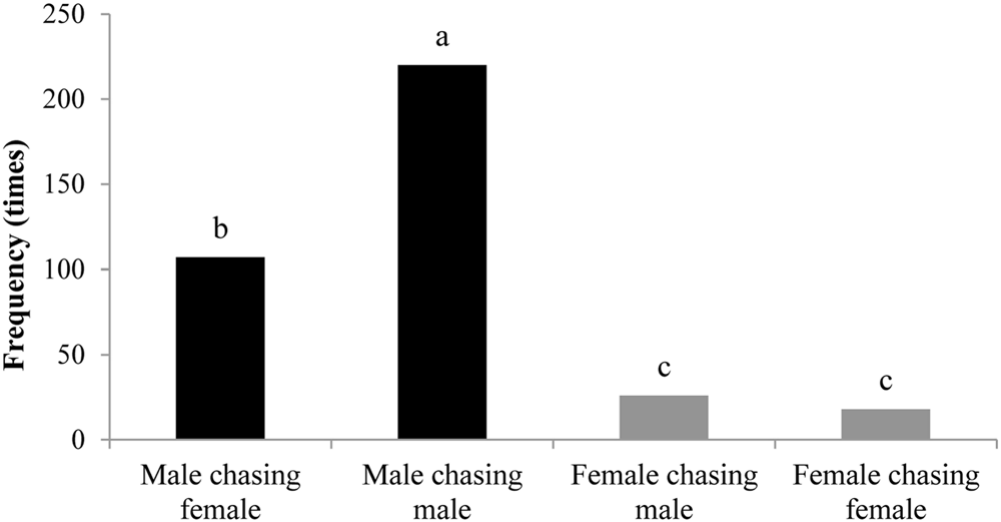
The frequency of occurrence of the four categories of chase during courtship of *Cethosia cyane cyane*.

In natural populations of *C. cyane cyane*, males always took the initiative and females were passive during courtship. Four kinds of courtship behavior were observed: male chasing male (59.3%), male chasing female (28.8%), female chasing male (7.0%), and female chasing female (4.9%) (Fig. 1). The frequencies of males chasing males and males chasing females were significantly different (*χ* ^*2*^ = 39.049, df = 1, *P* < 0.0001), but there was no significant difference in the frequencies of females chasing males and females chasing females (*χ* ^*2*^ = 1.455, df = 1, *P* = 0.228).

#### Winged and printed paper butterfly models

**Figure 2:**
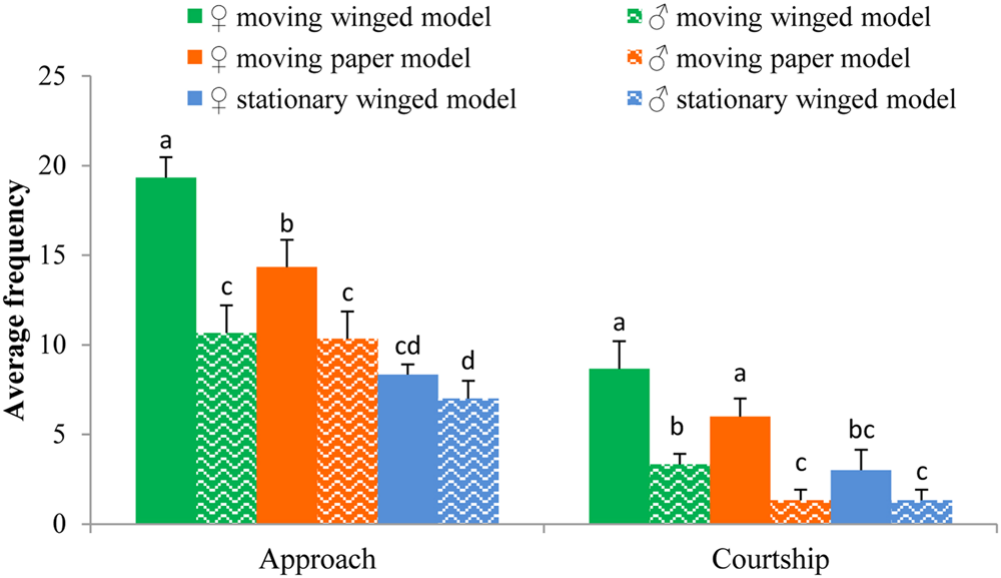
The average number of males approaching and courting moving and stationary winged and paper models. Different letters above bars indicate significantly different at *P* < 0.05 significance level.

The results showed that when adult odor was excluded, the courtship behavior of males chasing odorless winged and paper models differed from natural populations (Fig. 2). The average number of males approaching and courting female models (winged and paper models) was greater than for males approaching male models. The average number of males approaching and courting female winged models was significantly higher than for the male winged models (Z = −1.993, *P* = 0.046; and Z = −1.993, *P* = 0.046, respectively). Similarly, males preferably approached (Z = −1.964, *P* = 0.049) and courted (Z = −1.993, *P* = 0.046) female paper models than that of male’s. These results indicated that males could distinguish between the sexes during courtship relying on visual cues alone. The average number of males approaching and courting the winged models was greater than the paper models, suggesting that the differences between them were visible to males. The average number of males approaching (Z = −2.023, *P* = 0.043) and courting (Z = −1.993, *P* = 0.046) the moving and stationary models differed significantly, indicating that movement presents a further stimulus to chasing males.

#### Differences in wing size

*C. cyane cyane* is a sexually dimorphic insect and the wings of males and females differ in size. The forewings of males and females were 3.82 ± 0.19 cm and 4.09 ± 0.27 cm long, respectively. The hindwings of males and females were 2.90 ± 0.15 cm and 3.15 ± 0.22 cm long, respectively. The differences between male and female forewing length and hindwing length were significant (*P* = 0.001 and *P* < 0.0001, respectively).

**Figure 3:**
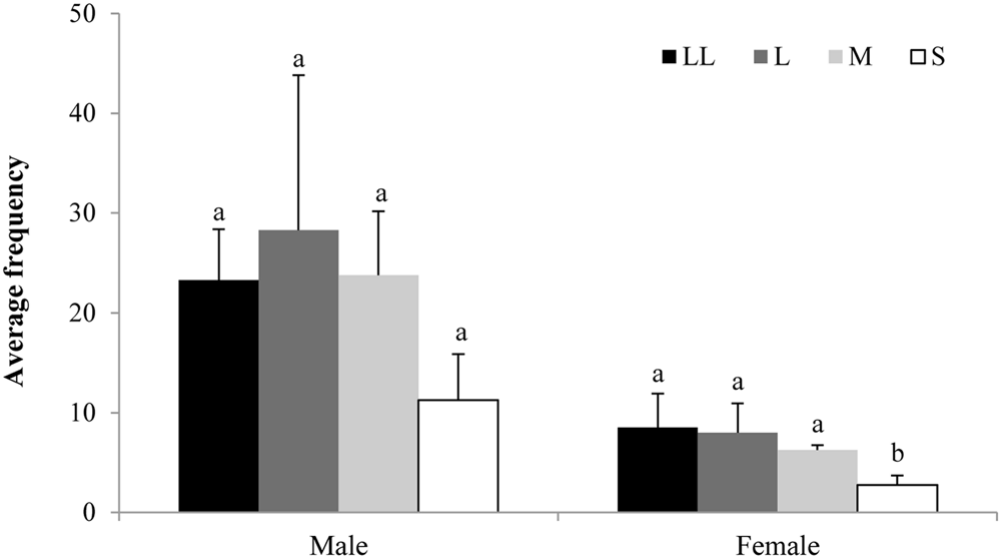
The number of male and female visits to four different sized female models. LL: female 1^1/2^ times normal size (wing span 9.84 cm); L: normal sized female (wing span 6.56 cm); M: female reduced to the male size (wing span 6.03 cm), and S: female ^1^/_2_ normal size (wing span 3.28 cm).

We used four different sizes of female printed paper models to attract males and females during courtship, and the results showed that more males and females visited the three larger models than smallest one, on average (Fig. 3). There was no significant difference in the number of males visiting the four different sizes of female models (one-way ANOVA, *P* = 0.104), indicating that males do not use the wing size difference between males and females to identify a partner.

### Wing color and color taxis

#### Differences between wings viewed under sunlight and ultraviolet (UV) light

**Figure 4:**
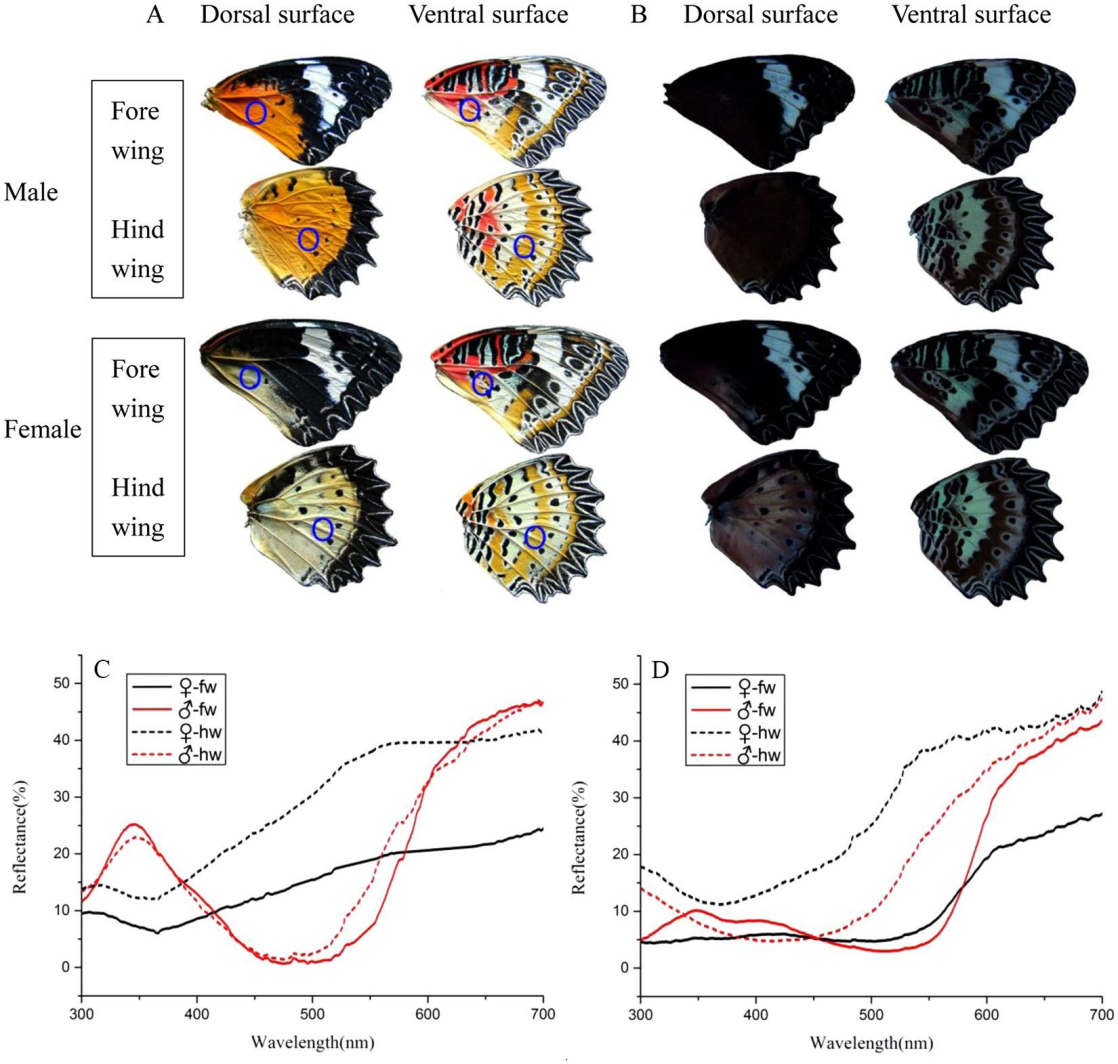
The wings of *Cethosia cyane cyane*. (**A**) Photographed under sunlight; (**B**) Photographed under UV light; (**C**) Reflectance spectra of the dorsal wings of male  and female; (**D**) Reflectance spectra of the ventral wings of male and female. fw: forewing, hw: hindwing. Circles on the wings depict the portion subjected to spectrophotometric analysis.

*C. cyane cyane* is a medium-sized butterfly and is sexual dimorphic in wing coloration. Males and females showed large differences in the dorsal coloration of both the forewing and hindwing, but little difference in the ventral coloration. The postmedian forewing and discal area of the hindwing is red-orange in males, but faint yellow in females. A single oblique transverse band appears in the subterminal region of the forewing in both sexes (Fig. 4A). The UV reflectivity of the white stripes was strong, with no difference between males and females. And the UV reflectivity of the ventral hindwings was also strong, with little difference between males and females. However, there was a large difference in the UV reflectivity of the dorsal hindwings, females reflecting more UV light than males (Fig. 4B), indicating that males might distinguish the gender of conspecifics using these UV reflectance differences.

#### Differences in the reflectance spectra of the wings

The reflectance spectra of the dorsal and ventral wing surfaces varied greatly in certain wavelength ranges between males and females (Fig. 4C,D). There were large differences in the reflectance spectrum of the dorsal forewing in the 350–370 nm (UV), 450–550 nm (cyan and green), and 600–700 nm (yellow, orange and red) ranges. There were large differences in the reflectance spectrum of the dorsal hindwing between males and females in the 350–370 nm and 450–550 nm ranges, and the ventral forewing in the 600–700 nm range.

#### Color taxis

**Figure 5:**
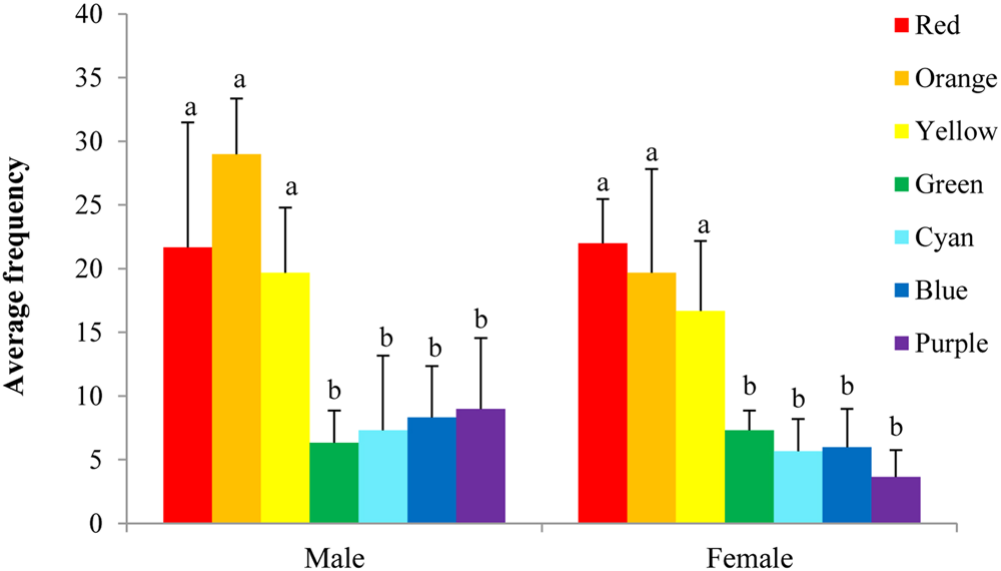
The number of male and female visits to seven butterfly-shaped cut-outs. Different letters above bars indicate significantly different at *P* < 0.05 significance level.

We used seven butterfly-shaped paper cut-outs to attract males and females. Both males and females were more likely to visit yellow, orange and red cut-outs, than green, cyan, blue, and purple ones (Fig. 5). Both males and females showed significant differences in the number of visits to the seven colored paper cut-outs (one-way ANOVA, ^♂^ *P* = 0.001; Kruskal–Wallis test, ^♀^ *P* = 0.012), indicating that males and females are more sensitive to long-wavelength light (red, orange, and yellow).

### Chemical information and behavioral assays

#### Chemical information

A total of 21 volatiles were detected in the bodies of adult *C. cyane cyane*. These included: alkanes, alkenes, alkynes, alcohols, ethers, terpenes and heterocyclic compounds. The most abundant volatiles were the alcohols (both males and females >40% of the total volatiles produced), followed by alkenes (>24%) and terpenes (>9%), with ethers the lowest at only about 0.2%. The terpenoids exhibited the greatest number of different compounds (seven), while the ethers were represented by only a single compound. Males and females showed little difference in the range of compounds produced. Twenty volatiles were observed in males, whereas 21 were observed in females, with cedrol being detected only in females (Table 1, Supplementary Fig. S1).

**Table 1:** Chemical volatiles recovered from adult males and females of *Cethosia cyane cyane*.

Peak No.	Compound	Female	Male
1	2-methyl-1-propene	+	+
2	2,2-dimethyl-1-propanol	++	++
3	α-pinene	+	+
4	2-methyl-2-propen-1-ol	+	+
5	β-pinene	++	++
6	ethylene glycol diallyl ether	+	+
7	3,5-dimethyl-1-hexene	+++	+++
8	3-phenyl-1-propyne	++	++
9	β-ocimene	+	++
10	trans-linalool oxide	+	++
11	cis- linalool oxide	+	+
12	linalool	++	++
13	2-(2-butoxyethoxy)-ethanol	+++	+++
14	unidentified	+	++
15	dihydroedulan II	++	++
16	edulan I	+	+
17	2,6,10-trimethyl-dodecane	+	+
18	tetradecane	+	+
19	hexadecane	+	++
20	cedrol	+	0
21	heneicosane	+	++

Significance levels: + = 0.01–1%, ++ = 1–20%, +++ = More than 20%.

#### Electrophysiological responses to eight volatile organic compounds

As shown above, there was little difference in either the types or quantities of volatiles produced by males and females. EAG experiments were performed to determine whether or not olfactory information might be involved in the identification of the opposite sex. Our results are summarized in Table 2. There were no significant differences between the EAG responses of males and females to low concentrations (1 μg/μl) and high concentrations (100 μg/μl) of hexadecane, 2-methyl-1-propene, 3-phenyl-1-propyne, β-pinene, 2-(2-butoxyethoxy)-ethanol, but significant response differences between males and females to low and high concentrations of cedrol, heneicosane and linalool oxide. The EAG responses to high concentrations of cedrol were significantly different between females and males (*P* = 0.033), but there were no significant differences between their responses to heneicosane and linalool oxide either at low or high concentrations (*P* > 0.05). Therefore, we speculated that cedrol might be involved in the precise identification of females by males.

**Table 2:** Electroantennogram (EAG) responses (Mean ± SD) of *Cethosia cyane cyane* to a range of doses of eight compounds.

Compound	♀		♂	
	1 μg/μl	100 μg/μl	1 μg/μl	100 μg/μl
hexadecane	1.67 ± 0.20Ab	1.47 ± 0.42Abc	1.50 ± 0.80Ab	2.08 ± 0.98Acd
2-methyl-1-propene	1.11 ± 0.51Ab	1.03 ± 0.38Ac	1.01 ± 0.13Ab	1.28 ± 0.44Ad
3-phenyl-1-propyne	1.71 ± 0.76Ab	1.74 ± 0.55Abc	1.52 ± 0.71Ab	1.51 ± 0.56Acd
β-pinene	1.73 ± 0.70Ab	2.23 ± 0.85Abc	1.66 ± 0.49Ab	2.04 ± 0.74Acd
2-(2-butoxyethoxy)- ethanol	1.98 ± 0.50Ab	2.63 ± 0.40Ab	2.23 ± 0.09Ab	2.79 ± 0.63Abc
cedrol	1.91 ± 0.32Cb	2.51 ± 0.56Bb	2.18 ± 0.41BCb	3.20 ± 0.39Ab
heneicosane	1.24 ± 0.19Bb	2.96 ± 0.82Ab	1.29 ± 0.26Bb	3.53 ± 0.37Ab
linalool oxide	3.23 ± 0.82Ba	7.72 ± 2.02Aa	3.14 ± 0.53Ba	6.61 ± 1.06Aa

Capital letters beside the data show the results of crosswise comparisons, and small letters show the results of lengthwise comparisons; the same letter indicates no significant difference (*P* > 0.05), and different letters indicate significant differences (*P* < 0.05).

#### Behavioral assays of olfactory cues

**Figure 6:**
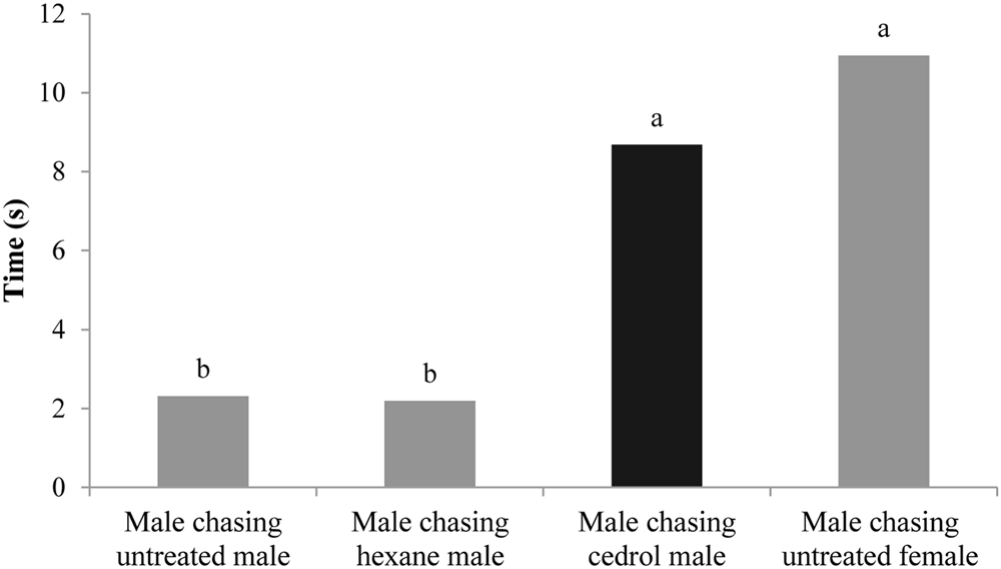
The average single chase time in experiments to assess the effect of cedrol. hexane male: male with wings painted with hexane; cedrol male: male with wings painted with cedrol. Different letters above bars indicate significantly different at *P* < 0.05 significance level.

To explore whether cedrol is the chemical cue used by males to identify females during courtship, we conducted behavioral validation experiments on males. Results showed that the chase time for the four groups was different. The average single chase time was: untreated females (10.95 s, N = 21) > males with wings painted with cedrol (8.68 s, N = 44) > untreated males (2.32 s, N = 120) > males with wings painted with hexane (2.20 s, N = 50) (Fig. 6). There were significant differences between the average single chase time of males chasing untreated males, males chasing males with wings painted with hexane, and males chasing males with wings painted with cedrol (*χ* ^*2*^ = 7.538, df = 2, *P* = 0.023). There was no significant difference between the average single chase time of males chasing males with wings painted with cedrol and males chasing untreated females (*χ* ^*2*^ = 0.200, df = 1, *P* = 0.655), indicating that cedrol is involved in the process of male recognition of females.

## Discussion

When butterflies mature to adulthood, their primary task is to copulate. A major problem before mating is the successful identification of an opposite sex individual in order to achieve effective copulation. There are many ways to identify a conspecific mate during courtship: mainly by visual cues^2, 3, 7^; mainly by olfactory cues^13, 21^; mainly by auditory cues^14^; mainly by tactile cues^22^; and by both visual and olfactory cues^5^. Our experiments show that males invariably take the initiative during courtship. There were significant differences in the average number of *C. cyane cyane* males approaching and courting females and males when presented with both winged models and paper models, indicating that males could distinguish the gender of conspecifics using visual cues alone. Cedrol was only found in female bodies and when applied to the wings of males it significantly prolonged the male chasing and recognition time. This may be because males mistook painted males for females or because the combination of male wing color with female odor confused the chasing males. Behavioral assays of olfactory cues suggested that cedrol is involved in the process of recognition of females by males at close range. Therefore, we conclude that male *C. cyane cyane* use both visual and olfactory cues to distinguish females during courtship. Visual cues play a major role in attracting males at the beginning of the courtship chase, while olfactory cues play a role in accurately identifying partners at close range.

In the natural population of *C. cyane cyane*, males chasing males was the most common case. But when we used winged and paper models to remove odor cues, males were more likely to visit the female models, indicating that adult odors might affect mate selection. The number of males chasing males was far greater than males chasing females during courtship, perhaps because males seek to remove the competition to secure mating rights^23, 24^. We have often observed male–male aggressive behavior (aggression accounted for 15% of the total number of chases) during courtship. However, when presented with odorless models, some males still chose to pursue other males. It may be that males identify males from a distance using wing color, while at close quarters they rely on odor cues for female recognition. It is also possible that individuals within the population differ in their ability to discriminate color.

The large differences in the reflectance spectra between male and female wings and color preference experiments illustrate the importance of visual cues in recognition of the opposite sex. The dorsal color of male and female wings differ greatly (male orange yellow, female faint yellow), as does their UV reflectance. Males and females differed in the reflectance spectra in the 350–370 nm, 450–550 nm and 600–700 nm ranges. Behavioral assays showed that males and females were more sensitive to long-wave light (red, orange, and yellow), and less sensitive to green, cyan, blue, and purple. This may provide a visual basis for discriminating the sexes. In this species, females are larger than males. However, there was no significant difference in the number of males visiting the four different sized female models, indicating that males do not use wing size difference to identify partners, but rely mainly on wing coloration to identify the opposite sex.

Flapping is important in attracting males to begin courtship. For example, the attractiveness of *Argynnis paphia* L. models to males increased proportionally to the speed of fluttering^4^. We found that fluttering winged models attracted more males to approach and court than did stationary ones. It appears that wing vibration is a highly visible sensory signal, and that butterflies are more sensitive to moving objects^25^. Another explanation is that males feel the vibration of the air produced by fluttering wings. To test the latter hypothesis, we connected male antennae to EAG recorders and used three different flow rates (17 ml/s, 20 ml/s, 25 ml/s) of moist air (to simulate different frequencies of butterfly flight) to stimulate the males’ antennae. Results showed that strong EAG responses were elicited by an air flow rate of 25 ml/s (EAG = 0.1857 ± 0.0275 mV). The EAG value at 25 ml/s was significantly higher than at both 17 ml/s (0.0667 ± 0.0170 mV) and 20 ml/s (0.0892 ± 0.0295 mV) (Wilcoxon signed-rank test, Z = −2.309, *P* = 0.021). Although we do not know the natural fluttering frequency of female *C. cyane cyane* during courtship, the results suggest that males may feel the air vibration caused by moving wings, and that it may play a certain role in attracting males. However, whether the male and female flap frequency could be used to identify the opposite sex has yet to be tested.

In many species of butterfly, males are more colorful and boldly patterned than females, especially on the dorsal wing surface. We also found that the dorsal wing surfaces of *C. cyane cyane* males are more brightly colored than females. Darwin^23^ first suggested that bright, male-specific coloration evolved in response to female mate preferences. Another explanation for bright, male-specific coloration is that it is used by males to discriminate females from males and perhaps even to assess the quality of potential competitors^21^. In addition, mathematical models have suggested that bright male coloration in insects generally could have evolved as a way of avoiding harassment by other males^26^.

In summary, *C. cyane cyane* males depend on both visual and olfactory cues to distinguish the sexes during courtship. In sexually dimorphic butterflies, strong differences in wing color are conducive to rapid identification of the opposite sex by males. The large sexual differences in color and small differences in volatiles may be common in butterflies as a result of natural selection. The limits imposed by small sexual differences in chemical cues leads to poorly developed olfaction and promotes the evolution of improved vision. Male identification of the opposite sex using differences in wing color or ultraviolet reflection requires further research and analysis. This species gives different weight to visual versus olfactory cues during feeding visits to flowers, with olfaction being given priority over vision^19^. However, males distinguish the sexes during courtship using both visual and olfactory cues. Whether the use of two kinds of signal is more effective than a single signal, and more conducive to reproductive success needs to be further explored.

## Materials and Methods

No specific permits were required for the field studies described.

### Research site

Experiments took place at the experimental station of the Research Institute of Resources Insects in Yuanjiang County, Yunnan Province, China (102°00′E, 23°36′N) in August 2014. The elevation is 400 m, with an average annual temperature of 19–20 °C, and an average annual rainfall of 500–600 mm.

### Experimental animals

*Cethosia cyane cyane* individuals were bred artificially at the experimental station. Larvae were raised on young leaves of *Adenia cardiophylla* (Mast.) Engl (Passifloraceae) under controlled conditions at 27 ± 2 °C with a 13:11 (L:D) cycle, and 60% relative humidity. The adults used for the chemical volatiles assay and electroantennogram (EAG) recordings were 2–3 days old, while those used for behavioral assays were 4–5 days old (males and females were allowed to mate on the second day after eclosion). Only virgin adults were used in the behavioral assays.

### Observation of courtship behavior

#### Natural population

Twenty sexually mature, unmated adults (♀:♂ = 1:1) were placed in a net enclosure (8 × 4 × 4 m), with an even distribution of eight nectar-producing plants (*Lantana camara* L.) and quantities of rotting fruit. Their courtship behavior was observed from 08:00 to 18:00 hrs. The occurrence of four activities was recorded (male chasing male, male chasing female, female chasing male, and female chasing female).

#### The winged models and printed paper models

**Figure 7:**
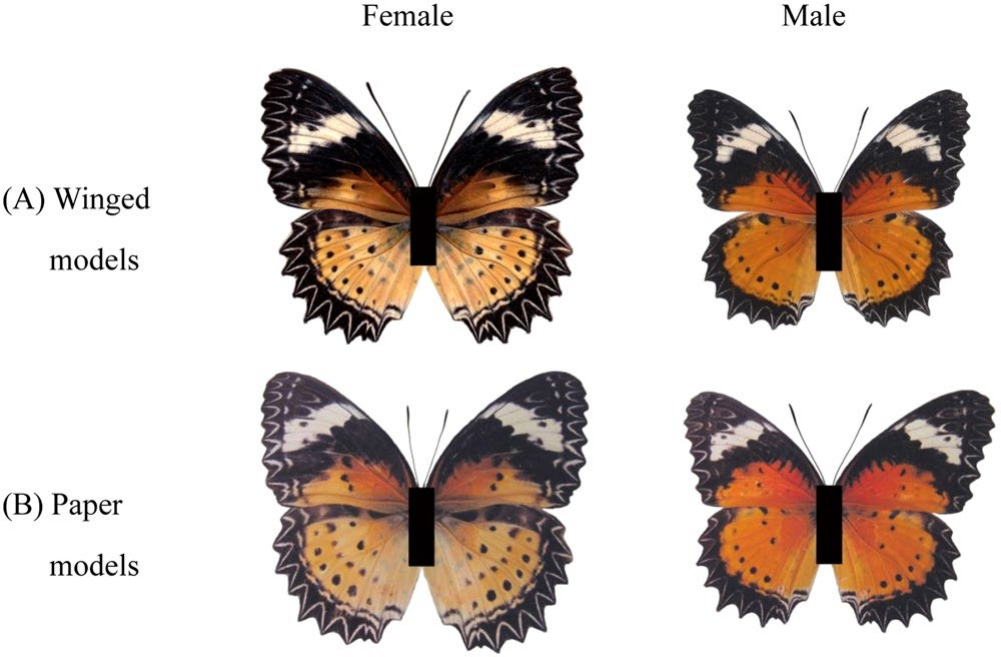
The winged and printed paper models of male and female *Cethosia cyane cyane*.

To test whether male responses were to wing color pattern, rather than pheromones, we placed 15 males in a 4 × 2 × 2 m insectary with models, with either dissected natural wings (♀ and ♂; Fig. 7A) or printed paper wings attached (♀ and ♂; Fig. 7B). The models were hung from a length of flexible wire in the center of a 60 cm diameter sphere and demarcated by a bamboo cross, and were either stationary or moving to simulate *C. cyane cyane* flight. Entry to the sphere was recorded as an ‘approach’, and sustained fluttering directed at the models as ‘courtship’^27, 28^. Three replicated 20 min observation periods (male responses to flapping and stationary models) were carried out per trial.

The reflectance spectra of printed paper models (see Supplementary Fig. S2) were analyzed using a spectrophotometer (Ocean Optics, Dunedin, Florida, USA).

#### Wing size

We made four different sizes of female paper models to examine whether males used the difference in wing size between males and females to identify the opposite sex: female 1^1/2^ times normal size (wing span 9.84 cm, LL), normal sized female (wing span 6.56 cm, L), female reduced to the male size (wing span 6.03 cm, M), and female ^1^/_2_ normal size (wing span 3.28 cm, S). Two of each of the four models (LL, L, M, S) were evenly spaced in the net enclosure, hung at a height of 1.8 m. At the beginning, 40 virgin males (or females) were placed in the net enclosure, and four replicate 20 min observation periods were performed. The positions of the models were changed in each replication. The frequencies of male (female) visits to the four types of model were recorded.

### Color response and wing reflectance spectra

#### Wing length and reflectance spectra

The forewing and hindwing length of 20 male and 20 female *C. cyane cyane* were measured. The dorsal and ventral surfaces of the forewing and hindwing were then stuck onto a black board, and photographed under normal sunlight using a Canon G10 camera. UV color determinations were performed using an ultraviolet spectrometer (WD-9403C, Beijing Liuyi Co., Beijng,China; with peak wavelength of 365 nm). We subsequently quantified the reflectance of male and female color markings using an Ocean Optics USB-2000 + spectrometer (170 ms integration time, 30 averaged scans), PX-2 pulsed xenon light source and magnesium oxide (MgO) reflectance standard. Reflectance was captured from a circular region 5 mm in diameter, with the wing rotated on a dual-axis stage to locate the orientation providing maximum brightness according to well-established protocols^29, 30^.

#### Color response

To quantify the sensitivity of males and females to different colors, we cut seven colored (red, orange, yellow, green, cyan, blue and purple) paper models the same size and shape as the butterfly. Two of each of these models were suspended, evenly spaced, in the net enclosure. Fifty males (females) were placed in the net enclosure, and three replicate 20 min observation periods were performed. The positions of the models were randomly changed for each replication. The frequencies of male (female) visits to the seven types of model were recorded.

### Volatile organic compounds (VOCs) and olfactory cues

#### Analysis of VOCs

The volatiles emitted from eight males and eight females were sampled using the solid-phase microextraction (SPME) technique and analyzed by gas chromatography and mass spectrometry (GC-MS, Thermo Fisher Scientific, Bellefonte, USA). To collect the odor emitted by males and females, we used a conical flask (height: 15 cm, diameter: 3 cm, V: 300 ml) with one small opening and one SPME fiber holder placed in the opening. The sample time was 1 h for the fiber and either eight males or eight females were kept together in the conical flask each time.

Polydimethylsiloxane-divinylbenzene (PDMS/DVB) fibers (65 μm, Supelco, Bellefonte, PA, USA) were used to sample the volatiles from live butterflies^31^. The SPME fibers were desorbed before each sampling by heating in a GC injector (250 °C for 10 min) with a He gas flow. Analyses were conducted using a Thermo Fisher TRACE GC ULTRA coupled to a Thermo Fisher ITQ 900 MS. A TR-1MS column (internal diameter: 0.25 mm, film thickness: 0.25 μm, length: 30 m) was used, programmed for 40 °C for 2 min then increased to 120 °C for 2 min (4 °C/min), and then increased to 230 °C for 5 min (5 °C/min) with an injector temperature of 250 °C, and He carrier gas at 69 kPa. Identification of the compounds was made by comparison of retention times and mass spectra with authentic reference samples.

#### EAG Recordings

The EAG techniques used in this study were similar to those described previously^32^. Glass capillaries (1.1 mm I.D.) filled with saline solution (4.7 mM KCl, 1.9 mM CaCl_2_, 130 mM NaCl) were used as electrodes. Antennal preparations were made by first cutting the base and distal end of the antenna with a scalpel. Two dilutions (1 and 100 μg/μl) of eight compounds (hexadecane, 2-methyl-1-propene, 3-phenyl-1-propyne, β-pinene, 2-(2-butoxyethoxy)-ethanol, cedrol, heneicosane, linalool oxide) were prepared in hexane (the control stimulus). These eight volatiles were selected for analysis because both sexes produced them in abundance, but in different quantities. An aliquot (20 μl) of each solution was applied to a piece of filter paper (10 × 30 mm). The stimulation was delivered at a flow rate of 10 ml/s in a 0.2 s puff using a stimulation device (Syntech). Antennae from five male and five female butterflies were used.

#### The cedrol attraction experiment

Following the results of the male and female EAGs, we painted the hindwings of six males with 10 μl cedrol (1/100 dilution in hexane, ca. 98 µg/individual). The control groups were: untreated males, males with hindwings painted with 10 μl hexane, and untreated females. Each of the four groups (with six individuals in each group) was placed in the net enclosure with 14 normal males and the time that the normal males spent in courtship chasing each experimental group was recorded.

### Statistical analyses

The data were analyzed using SPSS 18.0. Student’s *t*-test was used to determine significant differences in forewing and hindwing length between males and females, and the EAG values of individual volatiles of both sexes. Duncan’s multiple comparison tests were used to examine significant differences in EAG values of eight volatiles of females and males. The Wilcoxon signed-rank test was used to compare the number of approaches and courtship events between males and females. The Chi-square test was used for the other tests of difference.

## Electronic supplementary material

Supplementary Information
