# Supplementary material for: Role of visual and olfactory cues in sex recognition in butterfly Cethosia cyane cyane
Source: Sci Rep. 2017 Jul 10;7:5033. doi: 10.1038/s41598-017-04721-6 (PMC5504021; doi:10.1038/s41598-017-04721-6)
Supplement: Supplementary file 1 — Supplementary Information [file 41598_2017_4721_MOESM1_ESM.pdf]

Supplementary Information for:

Role of visual and olfactory cues in sex recognition in butterfly *Cethosia cyane cyane*

Chengzhe Li, Hua Wang, Xiaoming Chen*, Jun Yao, Lei Shi & Chengli Zhou

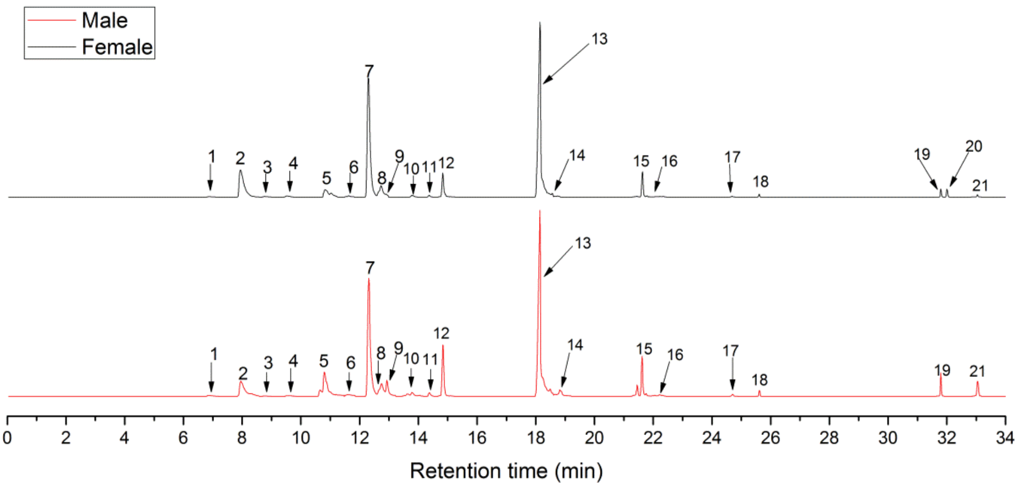

Figure S1. Total ion chromatograms of volatiles released by male and female *Cethosia cyane cyane*. A total of 21 volatiles were detected in the bodies of adults, with cedrol (20) only being detected in females.

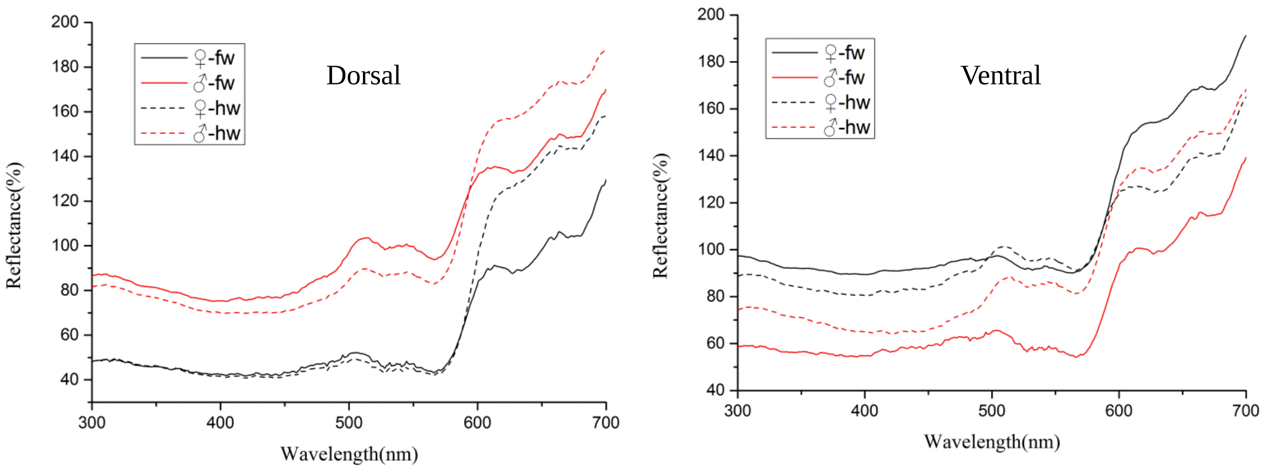

Figure S2. Reflectance spectra of colour patterns in paper models of male and female *Cethosia cyane cyane.* fw: forewing, hw: hindwing.

Fig. S2 shows the reflectance spectra of printed paper models are likely to differ from real wings in reflectance and wavelength in the dorsal forewing and hindwing in the 300–550 nm. But preferences can be replicated using printed paper models this provides confirmation that butterflies are indeed using color pattern as a cue and not some other aspect of our dissected wing models.
